# Hypothermia broadens the therapeutic time window of mesenchymal stem cell transplantation for severe neonatal hypoxic ischemic encephalopathy

**DOI:** 10.1038/s41598-018-25902-x

**Published:** 2018-05-16

**Authors:** So Yoon Ahn, Yun Sil Chang, Dong Kyung Sung, Se In Sung, Won Soon Park

**Affiliations:** 1 Samsung Medical Center, Sungkyunkwan University School of Medicine, Seoul, South Korea; 2 Department of Health Sciences and Technology, SAIHST, Sungkyunkwan University, Seoul, South Korea; 3 Stem Cell and Regenerative Medicine Institute, Samsung Medical Center, Seoul, South Korea

## Abstract

Recently, we have demonstrated that concurrent hypothermia and mesenchymal stem cells (MSCs) transplantation synergistically improved severe neonatal hypoxic ischemic encephalopathy (HIE). The current study was designed to determine whether hypothermia could extend the therapeutic time window of MSC transplantation for severe neonatal HIE. To induce HIE, newborn rat pups were exposed to 8% oxygen for 2 h following unilateral carotid artery ligation on postnatal day (P) 7. After approving severe HIE involving >50% of the ipsilateral hemisphere volume, hypothermia (32 °C) for 2 days was started. MSCs were transplanted 2 days after HIE modeling. Follow-up brain MRI, sensorimotor function tests, assessment of inflammatory cytokines in the cerebrospinal fluid (CSF), and histological evaluation of peri-infarction area were performed. HIE induced progressively increasing brain infarction area over time, increased cell death, reactive gliosis and brain inflammation, and impaired sensorimotor function. All these damages observed in severe HIE showed better, robust improvement with a combination treatment of hypothermia and delayed MSC transplantation than with either stand-alone therapy. Hypothermia itself did not significantly reduce brain injury, but broadened the therapeutic time window of MSC transplantation for severe newborn HIE.

## Introduction

Despite recent advances in neonatal intensive care, perinatal asphyxia and following hypoxic ischemic encephalopathy (HIE) still remain serious diseases with high mortality and neurologic sequelae in survivors, including epilepsy, mental retardation, learning disabilities, and cerebral palsy^1,2^. Currently, hypothermia treatment is the only available treatment that is known to be effective in improving the outcome of neonatal HIE^3–5^. However, even with hypothermia treatment, about half of HIE infants die or progress significant neurological complications^6,7^. In the severe type of HIE, outcomes are even more severe^6,7^. Therefore, the development of new, safe, and effective additional treatments besides therapeutic hypothermia, to enhance neuroprotective effects and improve the prognosis of severe neonatal HIE is an urgent requirement.

Recent studies reported the neuroprotective effects of mesenchymal stem cells (MSCs) transplantation in neonatal animal models of intraventricular hemorrhage (IVH)^8^, neonatal stroke^9^, and HIE^10–12^. We have also shown that concurrent treatment with hypothermia and intracerebroventricular MSC injection synergistically attenuates severe HIE in contrast to stand alone therapy^13^. Furthermore, phase I clinical trials that involved transplantation of autologous UCB mononuclear cells (MNCs) to neonates with HIE, in addition to the hypothermia treatment, or allogenic human UCB-derived MSCs to neonates with severe IVH^14^ have shown the treatments to be safe, feasible, and potentially efficacious. Overall, these data propose that cell based therapies combined with therapeutic hypothermia might act synergistically, and thus could be a novel effective therapy to improve the outcome of the currently intractable severe neonatal HIE.

Brain injury during neonatal HIE is an evolving process starting with a primary phase of hypoxic ischemic energy failure, followed by a latent phase of recovering cerebral energetics after resuscitation and a secondary phase of energy deterioration^15^. The existence of a time window (latent phase) following resuscitation enables the introduction of new therapies aimed to reduce the development of secondary energy failure. As the duration decreases, with increasing primary cerebral hypoxic ischemic insult^15^, the therapeutic time window of stem cell transplantation for severe neonatal HIE might be quite narrow^9,13,16^; therefore, administration closer to the time of primary hypoxic ischemic brain injury might result in better therapeutic outcomes. However, as an overnight thawing procedure of cryopreserved MSCs was necessary in our recently conducted phase I clinical trials for bronchopulmonary dysplasia^17^ and IVH (NCT02274428), the time between collection of UCB, arrival at the bedside, and initiation of the infusion ranged between 3.9 to 220 hours in the phase I clinical trial of autologous UCB transplantation for HIE^18^. Thus, it would be virtually impossible to apply both therapeutic hypothermia and stem cell transplantation simultaneously in clinical practice. Therefore, any treatment that could broaden the short therapeutic time window of stem cell transplantation would be clinically very useful. O’Brien *et al*.^19^ demonstrated that therapeutic hypothermia itself could prolong the therapeutic time window, and thus delay the start of secondary energy failure, in addition to its direct neuroprotection. In that case, by lengthening the therapeutic time window, we could first apply the only currently clinically available and known to be effective treatment—therapeutic hypothermia—and selectively apply additional therapies later, such as MSC transplantation only, in patients with severe HIE not responding to isolated hypothermia treatment, for further beneficial effects^20,21^.

In this study, we thus tried to determine whether hypothermia could broaden the therapeutic time window of delayed MSC transplantation for severe HIE in newborn rats. After approving severe HIE induced brain injury which involving >50% of the ipsilateral hemisphere using brain magnetic resonance imaging (MRI), the rat pups were randomly allocated to either of the experimental groups. The therapeutic effects of each treatment was assessed using *in vivo* serial brain MRI monitoring, sensorimotor function rotarod and negative geotaxis test, histological examination of the peri-infarct area by terminal deoxynucleotidyl transferase nick end labeling (TUNEL), and staining for glial fibrillary acidic protein (GFAP) and reactive microglial marker (ED-1).

## Results

### Serial Brain MRI and Injury Assessment

**Figure 1:**
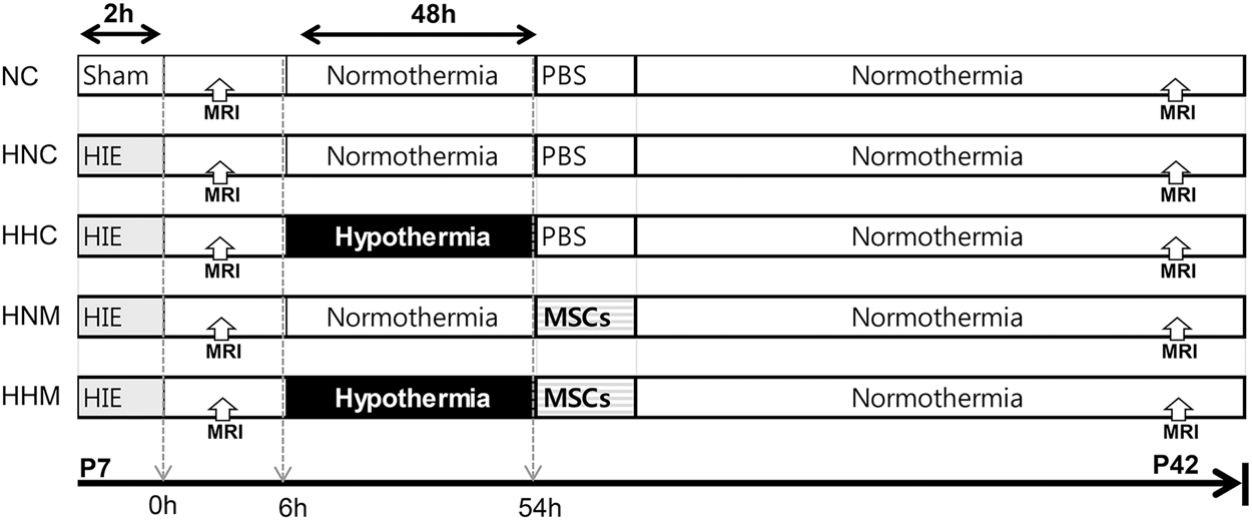
Experimental protocol.

**Figure 2:**
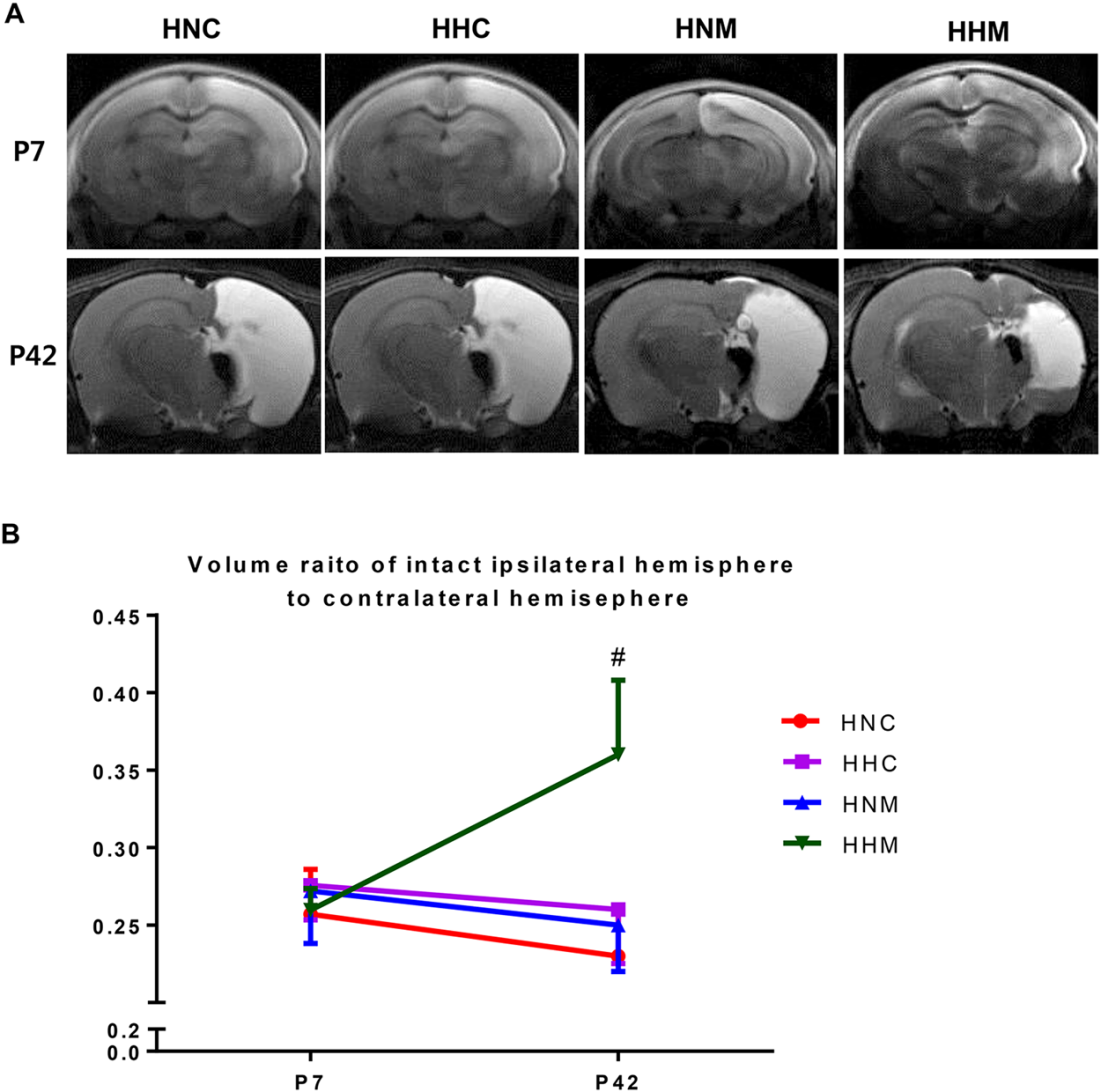
(**A**) Representative serial brain magnetic resonance images from treatment groups on P7 and P42. (**B**) Volume ratio of the ipsilateral intact area to the contralateral whole brain area measured by magnetic resonance imaging. Data are SEM. HNC, HIE + normothermia control; HHC, HIE + hypothermia; HNM, HIE + normothermia + MSCs; HHM, HIE + hypothermia + MSCs. ^#^*P* < 0.05 vs. HNC.

Figure 1 displays the experimental schedule and groups in the present study. Representative serial brain MRI obtained on P (postnatal day) 7 (2 h after HIE) and P42 (35 days after HI) in each experimental group are presented in Fig. 2A. Although the baseline ipsilateral intact brain volume evaluated on P7 was not significantly different between experimental groups, the intact brain volume in the HIE injury control group progressively reduced over time on follow-up brain MRI performed on P42 (Fig. 2B). The reduced intact brain volume observed in HIE control group rats was significantly attenuated in the combined treatment of hypothermia and delayed MSC, but not in the hypothermia or MSC single treatment.

### Cell Death and Reactive Gliosis

**Figure 3:**
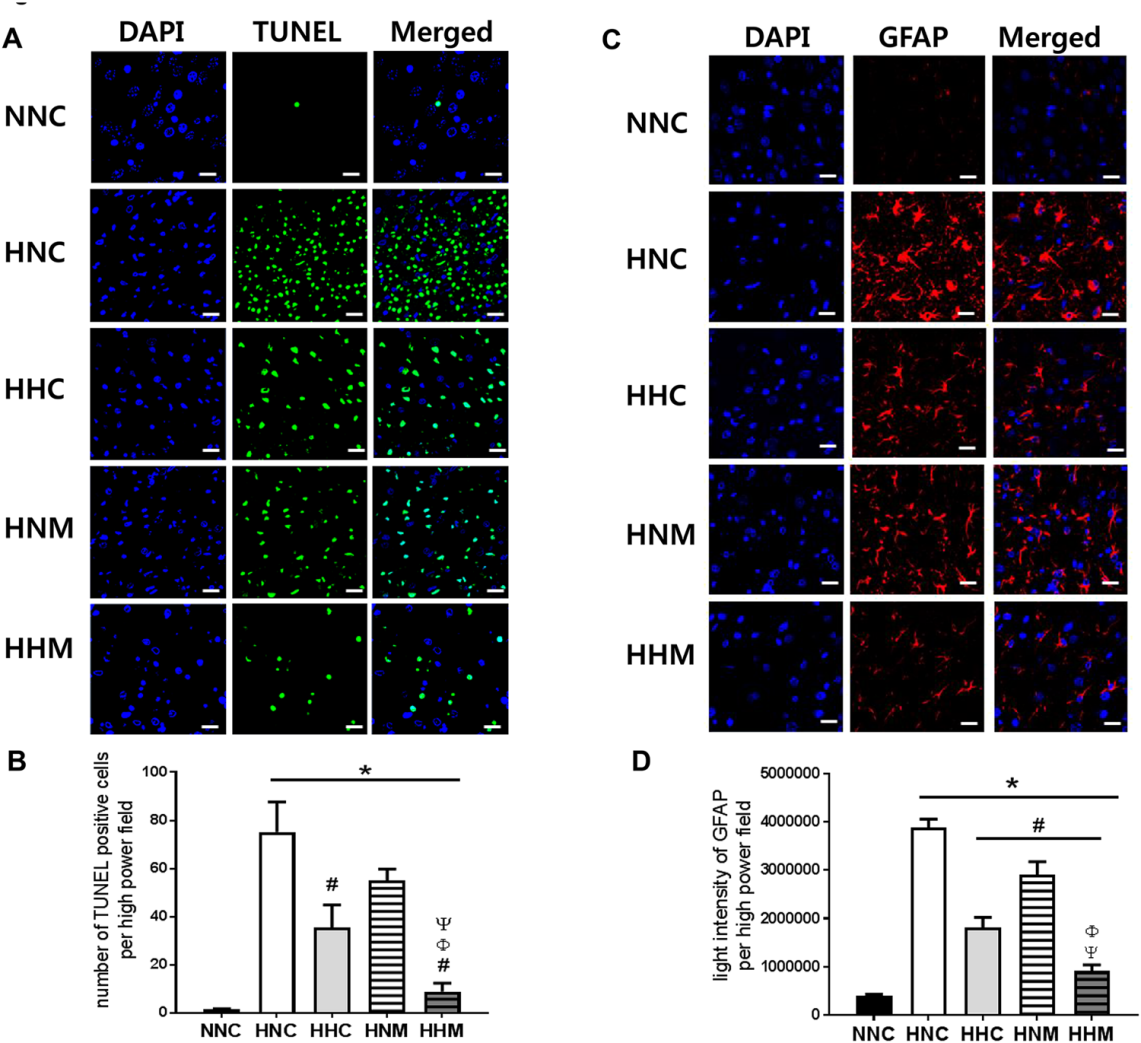
Representative immunofluorescence micrographs of the penumbra area with staining for terminal deoxynucleotidyl transferase nick end labeling (TUNEL) (**A**), glial fibrillary acidic protein (GFAP) (**C**), and DAPI (original magnification; x400, scale bars; 25 μm). Average number of TUNEL-positive cells (**B**) and average innsity of GFAP staining (**D**) in the penumbra area. Data are mean ± SEM. HNC, HIE + normothermia control; HHC, HIE + hypothermia; HNM, HIE + normothermia + MSCs; HHM, HIE + hypothermia + MSCs. ^*^P < 0.05 vs. NNC, ^#^*P* < 0.05 vs. HNC, ^Φ^*P* < 0.05 vs. HHC, ^Ψ^*P* < 0.05 vs. HNM.

Markedly raised number of TUNEL-positive cells was observed in all HIE groups compared to the normal control. HIE-induced increased cell death was significantly improved with hypothermia treatment, with better attenuation by the combined treatment of hypothermia and delayed MSC treatment than by hypothermia single treatment (Fig. 3A,B).

Elevated GFAP level, indicative of reactive gliosis, was observed in all the HIE groups, as compared with the normal control. This enhanced reactive gliosis in the HIE control group was significantly ameliorated by hypothermia and/or MSC treatment. Combined treatment of hypothermia and delayed MSC showed better improvement than hypothermia only or MSCs only treated group (Fig. 3C,D).

### Inflammation

**Figure 4:**
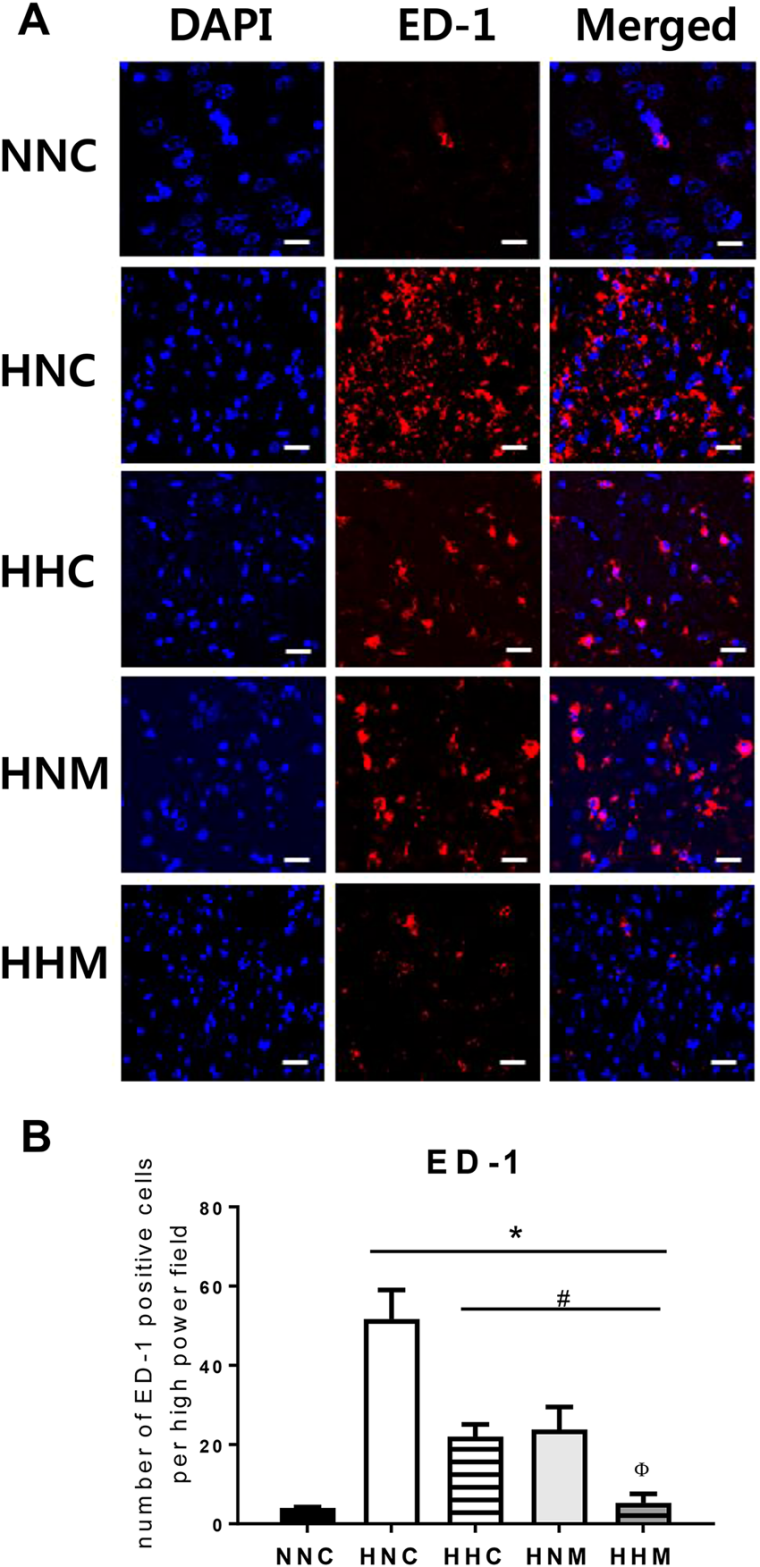
(**A**) Representative immunofluorescence micrographs of the penumbra area with staining for ED-1 (red) and DAPI (blue) (original magnification; x400, scale bars; 25 μm). Average optical density of ED-1 staining (**B**) in the penumbra area. Data are mean ± SEM. HNC, HIE + normothermia control; HHC, HIE + hypothermia; HNM, HIE + normothermia + MSCs; HHM, HIE + hypothermia + MSCs. ^*^P < 0.05 vs. NNC, ^#^*P* < 0.05 vs. HNC, ^Φ^*P* < 0.05 vs. HHC.

**Figure 5:**
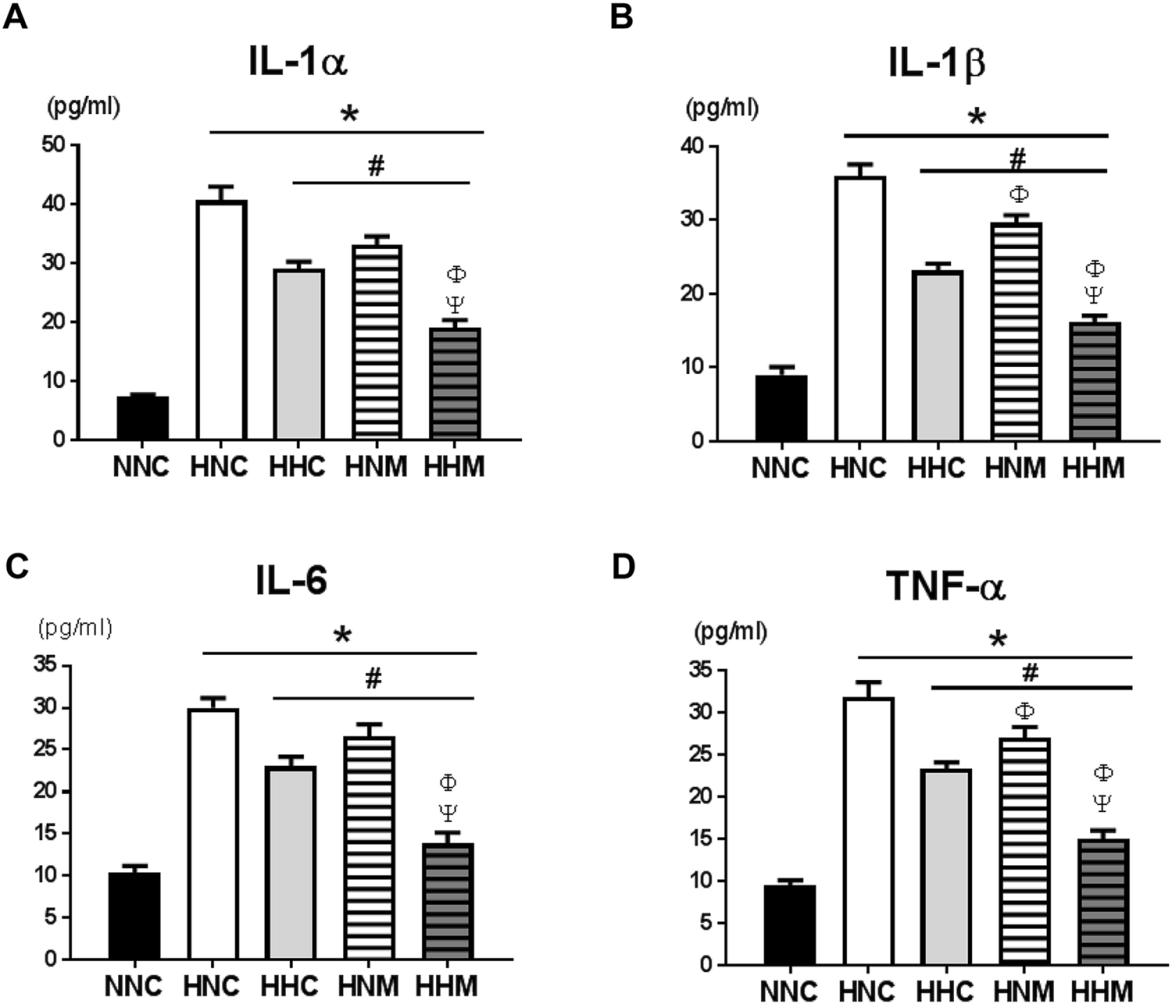
Concentration of inflammatory cytokines interleukin (IL)-1α, IL-β, IL-6, and tumor necrosis factor- α in cerebrospinal fluid on P42. Data are mean ± SEM. HNC, HIE + normothermia control; HHC, HIE + hypothermia; HNM, HIE + normothermia + MSCs; HHM, HIE + hypothermia + MSCs. ^*^P < 0.05 vs. NNC, ^#^*P* < 0.05 vs. HNC, ^Φ^*P* < 0.05 vs. HHC, ^Ψ^*P* < 0.05 vs. HNM.

Increased ED-1-positive microglial cells in the peri-infarct area were observed in all the HIE groups, as compared to the normal control group. This escalated number of activated microglial cells was significantly attenuated by hypothermia and/or MSC treatment (Fig. 4). Combined treatment of hypothermia and delayed MSC showed better improvement than hypothermia only or MSCs only treatment (Fig. 4). When compared to normal control, significantly elevated levels of inflammatory cytokines such as interleukin (IL)-1α, IL-1β, IL-6, and tumor necrosis factor (TNF)-α in the CSF obtained at P42 were observed in all HIE groups. These increased cytokine levels were significantly decreased by hypothermia and/or MSCs treatment (Fig. 5). Moreover, combined treatment group showed better attenuation than hypothermia only or MSC only treatment (Fig. 5).

### Functional Behavior Tests

**Figure 6:**
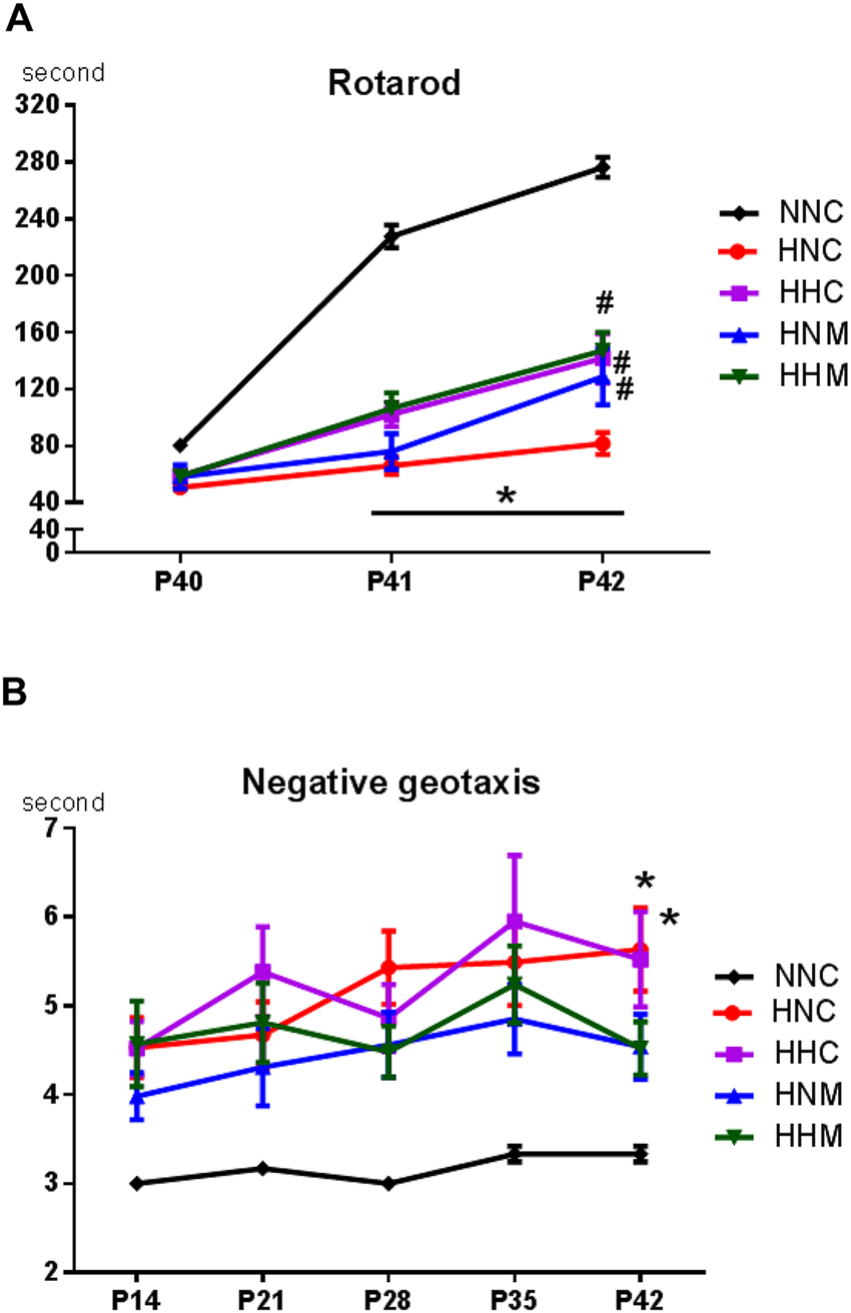
Sensorimotor functional outcomes on rotarod (**A**) and negative geotaxis test results (**B**). Data are mean ± SEM. HNC, HIE + normothermia control; HHC, HIE + hypothermia; HNM, HIE + normothermia + MSCs; HHM, HIE + hypothermia + MSCs. ^*^P < 0.05 vs. NNC, ^#^*P* < 0.05 vs. HNC.

To evaluate sensorimotor functions, rotarod test and negative geotaxis test were performed. In the rotarod test, the normal control group, demonstrated elongated latency to fall from P40 to P42, implying adequate learning curve over time. However, the HIE injury control group, presented significantly shorter latency to fall at P41 and P42 than normal controls (Fig. 6A). At P42, hypothermia and/or MSC treated groups, showed improvement in sensorimotor function without any difference between groups (Fig. 6A). In the negative geotaxis test, normal control group presented a short duration for rotating, indicating a quick response and intact sensorimotor function (Fig. 6B). The HIE controls displayed significantly longer duration, which indicated impaired function, than the normal controls (Fig. 6B). However, no statistical difference was observed among normal control group, MSCs single treatment group, combined treatment group (Fig. 6B).

## Discussion

In the present study, intraventricular route of MSC transplantation was extrapolated from findings in newborn rats with severe IVH showing better paracrine potency and therapeutic efficacy with local intraventricular transplantation than with systemic intravenous transplantation of MSCs^22^; moreover, a dose of 1 × 10^5^ cells was determined, based on the results of our previous studies showing significant neuroprotection with the same dose in newborn rats with severe neonatal stroke^9^ and concurrent hypothermia and MSC transplantation^13^. There are some reports that MSCs when given intravenously or intra-arterially can cause pulmonary embolism^23,24^ which can trigger the development of PPHN in the newborn infants. In the present study MSCs were given by local, intracerebroventricular administration, not by systemic intravascular administration which can avoid the possible occurrence of pulmonary embolism and subsequent PPHN.

In the present study, as the clinical conditions were quite lethargic due to induction of severe HIE involving more than half of the ipsilateral hemisphere, the analgesics/anesthetics were not routinely given to the rat pups during hypothermia treatment. However, there is a possibility that the absence of anesthesia/analgesia during hypothermia can cause the stress in rat pups and contribute to the failure of reducing the brain injury by TH alone^25^ as well as showing less effects by the combination of TH and MSC in the present study.

Although we observed better, robust neuroprotection with combined treatment of hypothermia and delayed MSC transplantation in the present study, further meticulous study for determination of the optimal route, dose, timing, and safety under hypothermia would be required for favorable translation of these experimental results into neonatal clinical practice.

Hypothermia treatment is a time bound critical emergency^26^, and its effective neuroprotective time window has been known to be within the first 6 hours after HI^27,28^. In the present and previous studies, hypothermia treatment at 32 °C, started 3–6 hours after HI, for 1–2 days failed to significantly attenuate severe HIE involving >50% of the ipsilateral hemisphere. These findings suggest that although hypothermia prolongs the therapeutic time window, direct neuroprotection by hypothermia itself, particularly against severe HIE, is neither quite significant nor persistent^19^. As current data in the present study demonstrate therapeutic hypothermia could broaden the therapeutic time window of MSCs transplantation for up to 2 days after severe HIE, after applying hypothermia first, we may determine MSCs transplantation later on selective cases not responding to therapeutic hypothermia in clinical practice. As our previous and present studies were neither designed to elucidate the optimal therapeutic time window, temperature and duration for hypothermic neuroprotection nor designed to investigate the relationship between primary HI insult severity and hypothermia efficacy, further studies would be necessary to clarify this.

Recently, we demonstrated that combined treatment of hypothermia and MSC transplantation synergistically improved severe HIE compared to single therapy^13^. In the present study, we observed that initial start of hypothermia treatment followed by delayed human UCB-derived MSC transplantation 2 days later better and robustly attenuated severe HIE-induced brain injuries such as progressively increasing brain infarction, CSF cytokine levels, apoptotic cells, microgliosis and astrocytosis, and impaired behavioral tests than either therapy alone. Considered together, these data may propose that hypothermia not only enhances therapeutic efficacy but also extends the therapeutic time window of human UCB-derived MSC transplantation for severe HIE. These results are quite encouraging for clinical translation, as we could first start the currently standard hypothermia treatment, and later on tailor stem cell transplantation only to individual neonates not responding to hypothermia treatment in order to fully optimize neuroprotection against severe HIE^20,21^.

We previously demonstrated that apoptosis plays a critical role in development of brain infarction, and reduce of apoptosis substantially decreased the ensuing brain infarction in the rat pup model of neonatal cerebral HI^29^. In the present study, an enhanced number of TUNEL-positive cells induced by severe HIE was significantly attenuated in all treatment, with best, robust attenuation in the combined hypothermia and delayed MSC transplantation group. Severe HIE-induced brain infarction was significantly improved only in the combined hypothermia and delayed MSC transplantation group. These findings suggest that hypothermia could broaden the therapeutic time window by preserving tissue viability and prolonging survival due to downregulation of the metabolic rate^30^, excitotoxicity^31^ and oxidative stress^32,33^, along with preservation of energy stores^19,34,35^, and anti-apoptotic effects;^3,36^ further it could potentiate the strong anti-apoptotic and the ensuing anti-infarct effects of delayed MSC transplantation^14^.

An enhanced GFAP level, representing increased astrocytic gliosis, is a specific biomarker for newborn HIE severity^37^. Therefore, GFAP could be useful for identifying newborn infants with HIE needing treatment, assessing treatment efficacy, and providing prognostic information. In the present study, although increased GFAP levels shown in the rats with severe HIE were significantly improved in all treatment groups, best improvement was observed in the combined hypothermia and delayed MSC transplantation group compared to the stand alone therapy groups. Our findings support the hypothesis that combined treatment of hypothermia and delayed MSC transplantation is superior to either therapy alone in severe neonatal HIE.

In neonatal HIE, the extent of inflammatory responses correlates with the severity of brain injury, and could thus predict neurological deficits in infants with HIE^38^. In the present study, while increased brain ED-1 positive microglia and levels of inflammatory cytokines in the CSF were significantly decreased in all treatment groups, the augmented inflammatory response was best attenuated with combined hypothermia and delayed MSC transplantation group compared with either therapy alone group. These data may suggest that the anti-inflammatory effects of stand-alone hypothermia or delayed MSC transplantation^3,39^ are not sufficient to block the severe HIE-induced brain injury, and only combined hypothermia and delayed MSC transplantation showed significant and best anti-inflammatory and neuroprotective effects against severe neonatal HIE.

Besides attenuating brain infarct area, improving behavior function is crucial for clinical translation of combined hypothermia and delayed transplantation of MSC for severe perinatal HIE. In the results of present study, although brain infarct volume significantly improved only in the combined hypothermia and delayed MSC transplantation group, severe HIE-induced impaired rotarod test results improved in all treatment groups, and negative geotaxis test results improved in delayed MSC transplantation with or without therapeutic hypothermia at P42. The results of rotarod test which performed during 3 consecutive days from P40–42, presented similar latency to fall in all groups on the first test day (P40). After repetitive trial during 3 days, normal group rats achieved appropriate learning curve but, HIE control group did not show any significant improvement. However, hypothermia and/or MSCs transplantation treatment improved this impaired learning capacity induced from HIE injury. Scholz *et al*.^40^ displayed rotarod test induced changes in brain structure using multimodal MRI and rotarod training was associated with increase of volume and fractional anisotropy in the hippocampus. In concert, these findings may suggest that besides intact brain volume, other factors such as the improved myelination or involvement of critical areas such as the hippocampus might be involved in sensorimotor function improvement. Furthermore, the improved behavioral function test results observed on P42 imply that neuroprotection of hypothermia and/or delayed MSC transplantation for severe neonatal HIE could persist into human adolescence^41^.

Combined treatment of hypothermia and human UCB-derived MSC transplantation best attenuated brain infarction and improved behavioral outcomes after severe HIE, when compared to either therapy in isolation. The neuroprotective action of combined therapy might be primarily related to the synergistic anti-apoptotic and anti-inflammatory effects. The results of the present study that hypothermia prolonging the therapeutic time window of MSC transplantation supports the notion that we could first provide therapeutic hypothermia and later selectively apply delayed MSC transplantation only to patients with severe HIE not responding to therapeutic hypothermia for further benefits. For successful clinical translation, more detailed preclinical investigations on the safety of the MSC transplantation to newborns with HIE are necessary.

## Materials and Methods

Institutional Animal Care and Use Committee of Samsung Biomedical Research Institute approved all experimental protocols of this present study, and we followed the institutional and National Institute of Health guidelines for laboratory animal care.

### Cell preparation

Human umbilical cord blood (UCB)-derived MSCs were purchased from Medipost Co., Ltd. (Seoul, Korea). MSCs from a single donor at passage 6 were used in this study.

### Animal model

To exclude sex-related differences in brain injury severity^42^, only male Sprague-Dawley rats (Orient Co., Seoul, Korea) on postnatal day (P) 7 were used. Figure 1 displays the experimental schedule. To induce cerebral hypoxia-ischemia (HI), right carotid artery was ligated and rat pups were exposure to 8% oxygen for 2 h as previously described^13^. Induction of severe HIE which involving more than 50% of the ipsilateral hemisphere volume was confirmed by DWI brain MRI done within 2 h after modeling. Then after we randomly allocated rat pups into four study groups: the HIE with normothermia control group (HNC, n = 11); HIE with hypothermia control group (HHC, n = 10); HIE with normothermia MSC transplantation group (HNM, n = 12); and HIE with hypothermia MSC transplantation group (HHM, n = 10). A normoxia normothermia control group rat pups received a sham operation (NNC, n = 5). Hypothermia intervention at a target temperature of 32 °C for 2 days^43^ was started 3 hours after modeling. During temperature intervention period, rectal temperature of pups was monitored (Supplementary Figure S1). After hypothermia treatment, 2 days after HI, MSCs (1 × 10^5^ cells in 10 µl saline) were administered. Follow-up *in vivo* brain MRI was done on P42. To assess sensorimotor function, rotarod and negative geotaxis test were conducted. Cerebrospinal fluid (CSF) sample was collected by cisternal tap to assess inflammatory cytokines. The rats were sacrificed, and brain tissue samples were preserved for histologic analyses on P42.

### Transplantation of donor cells with stereotaxis

For donor cell transplantation, 1 × 10^5^ human UCB-derived MSCs in 10 µl saline were administered into the ipsilateral right lateral ventricle using a stereotactic method (Digital Stereotaxic Instrument with fine drive, MyNeurolab, St. Louis, MO, USA; coordinates, x = +0.5, y = +1.2, z = −2.7 mm relative to bregma) at 2 days after HI^13^. For the HNC and HHC groups, an equivalent volume of saline was administered using the same method.

### Post-ischemic temperature modulation

For the temperature intervention, pups were separated from the mothers and placed in a temperature-controlled chamber for 24 h, separated by plastic containers. Ambient temperature inside the chamber was 34.0 °C for normothermia (HNC, HNM) and 31.0 °C for hypothermia (HHC, HHM). During the temperature intervention, temperatures were maintained at 35.5–36.5 °C for normothermia and 31–32 °C for hypothermia. Throughout the temperature intervention, pups were fed five times per day with 0.5 ml of milk formula, using a 22-G animal feeding needle.

### *In vivo* MRI assessment

Brain MRI was used to confirm severe baseline brain injury after HIE modeling on P7 and to monitor changes in damage on P42. MRI was performed using a 7.0-Tesla MRI system (Bruker-Biospin, Fällanden, Switzerland) as described previously^9,14^. Lesions were identified as hyperintense areas in DWI performed 2 hours after HI and hyperintense areas in T2-weighted imaging at P42. The intact ipsilateral-to-whole-contralateral hemispheric brain volume ratio was calculated as a measure of brain injury, as previously reported^9,13^. Volume estimates were made according to Cavalieri’s principle^9^. The investigator was blinded to the treatment group.

### TUNEL assay

Immunofluorescent terminal deoxynucleotidyltransferase-mediated deoxyuridine triphosphate nick end-labeling (TUNEL) (kit S7110 ApopTag, Chemicon, Temecula, CA, USA) was used to evaluate apoptosis in brain sections according to the manufacturer’s protocol as previously described^8^. Three coronal sections (+0.95 mm to −0.11 mm/bregma) were chosen from each brain, and three random nonoverlapping fields were selected in the cortical penumbra region from each section. The number of TUNEL-positive nuclei in selected fields were counted by an evaluator who was blind to the experimental groups.

### Immunohistochemistry

Immunofluorescence histochemical staining was performed for GFAP (rabbit polyclonal; Dako, Glostrup, Denmark) as an astrocytic glial marker and ED-1 (mouse monoclonal; Millipore, Concord Road, MA) as a marker for reactive microglia as previously reported. Three coronal sections (+0.95 mm to −0.11 mm/Bregma) were stained from each section, and three random nonoverlapping fields in the cortical penumbra area were selected from each section. The optical density of immunofluorescence in fields was measured using ImageJ software by an observer blind to the experimental groups (National Institutes of Health, USA).

### Enzyme-linked immunosorbent assay

Frozen CSF samples at −70 °C were thawed and centrifuged at 2,000 × *g* for ten minutes at 4 °C. Interleukin (IL)-1α, IL-1β, IL-6, and tumor necrosis factor (TNF)-α concentrations in CSF were measured using Milliplex MAP enzyme-linked immunosorbent assay (ELISA) kits according to the manufacturer’s protocol (Millipore, Billerica, MA, USA)^14^.

### Behavioral tests

To assess sensorimotor function, rotarod tests were performed at P40-P42 by analyzing the latency to fall. All animals were tested on three consecutive days. As rats can learn rotarod tests, the values were analyzed by date, and the latency to fall was used as previously reported^14^. Negative geotaxis test was performed at P12, 14, and 17. The time required for pups to rotate 180° to face uphill after release on a slanted slope was recorded. The values were analyzed by date, and the average time was used as final data. The evaluator was blind to the treatment groups.

### Statistical analysis

Data are expressed as mean ± standard error of the mean (SEM). For continuous variables, statistical comparison between groups was by one-way analysis of variance (ANOVA) and Tukey’s post hoc analysis. For time-course variables, repeated-measured ANOVA with Tukey’s post hoc comparison was performed. A p-value < 0.05 was considered significant. All data were analyzed using SPSS version 17.0 (IBM, Chicago, IL, USA).

## Electronic supplementary material

Supplementary information
