# Supplementary material for: Hypothermia broadens the therapeutic time window of mesenchymal stem cell transplantation for severe neonatal hypoxic ischemic encephalopathy
Source: Sci Rep. 2018 May 16;8:7665. doi: 10.1038/s41598-018-25902-x (PMC5955959; doi:10.1038/s41598-018-25902-x)
Supplement: Supplementary file 1 — Supplementary information [file 41598_2018_25902_MOESM1_ESM.docx]

Hypothermia broadens the therapeutic time window of mesenchymal stem cell transplantation for severe neonatal hypoxic ischemic encephalopathy

So Yoon Ahn, MD, PhD1,3, Yun Sil Chang, MD, PhD1,2,3, Dong Kyung Sung, PhD1,3, Se In Sung, MD, PhD1,3, Won Soon Park, MD, PhD1,2,3*

1Samsung Medical Center, Sungkyunkwan University School of Medicine, Seoul, South Korea

2Department of Health Sciences and Technology, SAIHST, Sungkyunkwan University, Seoul, South Korea

3Stem Cell and Regenerative Medicine Institute, Samsung Medical Center, Seoul, South Korea

So Yoon Ahn and YunSil Chang contributed equally as co-first authors.

Corresponding author: Won Soon Park, M.D., Ph.D

Department of Pediatrics, Samsung Medical Center; Sungkyunkwan University School of Medicine, 50 Irwon-dong, Gangnam-gu, Seoul 135-710, Korea

Tel: +82.2-3410-3523, Fax: +82.2-3410-0043

E-mail: wonspark@skku.edu, [ws123.park@samsung.com](mailto:ws123.park@samsung.com)


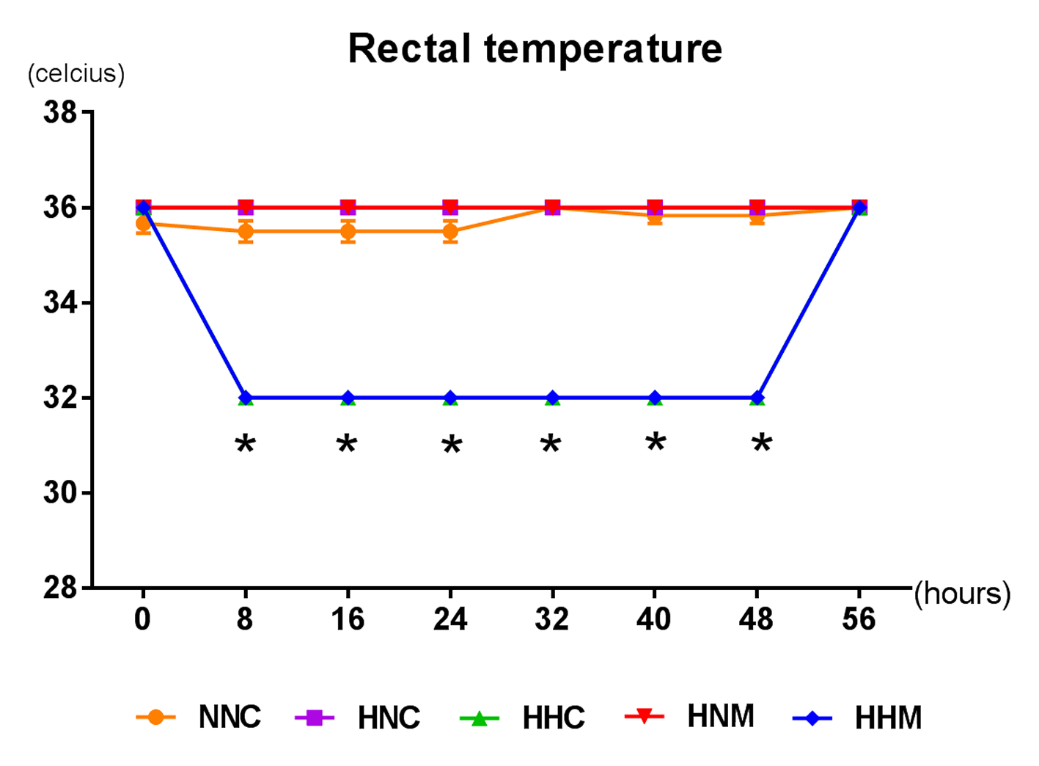


Figure S1. Rectal temperature in experimental groups. Temperatures in each group remained stable during the intervention and were significantly different between normothermia and hypothermia groups at each measurement. Data are mean ± SEM.

NNC, normal+normothermia control; HNC, HIE+normothermia control; HHC, HIE+hypothermia; HNM, HIE+normothermia+MSCs; HHM, HIE+hypothermia+MSCs. * P < 0.05 vs. normothermia groups (NNC, HNC, HNM).
